# Enriched Environment Modulates Sharp Wave-Ripple (SPW-R) Activity in Hippocampal Slices

**DOI:** 10.3389/fncir.2021.758939

**Published:** 2021-12-03

**Authors:** Lucie Landeck, Martin E. Kaiser, Dimitri Hefter, Andreas Draguhn, Martin Both

**Affiliations:** ^1^Institute of Physiology and Pathophysiology, Heidelberg University, Heidelberg, Germany; ^2^RG Animal Models in Psychiatry, Department of Psychiatry and Psychotherapy, Central Institute of Mental Health, Medical Faculty Mannheim, University of Heidelberg, Heidelberg, Germany

**Keywords:** hippocampus, sharp waves, ripples, plasticity, network oscillations

## Abstract

Behavioral flexibility depends on neuronal plasticity which forms and adapts the central nervous system in an experience-dependent manner. Thus, plasticity depends on interactions between the organism and its environment. A key experimental paradigm for studying this concept is the exposure of rodents to an enriched environment (EE), followed by studying differences to control animals kept under standard conditions (SC). While multiple changes induced by EE have been found at the cellular-molecular and cognitive-behavioral levels, little is known about EE-dependent alterations at the intermediate level of network activity. We, therefore, studied spontaneous network activity in hippocampal slices from mice which had previously experienced EE for 10–15 days. Compared to control animals from standard conditions (SC) and mice with enhanced motor activity (MC) we found several differences in sharp wave-ripple complexes (SPW-R), a memory-related activity pattern. Sharp wave amplitude, unit firing during sharp waves, and the number of superimposed ripple cycles were increased in tissue from the EE group. On the other hand, spiking precision with respect to the ripple oscillations was reduced. Recordings from single pyramidal cells revealed a reduction in synaptic inhibition during SPW-R together with a reduced inhibition-excitation ratio. The number of inhibitory neurons, including parvalbumin-positive interneurons, was unchanged. Altered activation or efficacy of synaptic inhibition may thus underlie changes in memory-related network activity patterns which, in turn, may be important for the cognitive-behavioral effects of EE exposure.

## Introduction

Complex nervous systems support experience-dependent learning, lending behavioral flexibility and individuality to animals. Such adaptive processes require lasting changes at the molecular, cellular, network, and system-wide levels. At the same time, much of the brain’s structure and function is genetically determined, posing the question of gene-environment interactions (GEI) in the formation of individually adapted neuronal mechanisms. The mechanisms and extent of gene-environment interactions have become a major topic in basic and clinical neurosciences (Ohline and Abraham, 2019; Rogers et al., 2019).

Animal studies on GEI rely strongly on comparisons between groups of animals raised or kept in different environments. A key paradigm is the so-called “enriched environment” (EE) approach in which animals (mostly rodents) are kept in extended cages supplied with different objects, devices facilitating complex movements, and conspecifics. Following earlier, unsystematic studies, pioneering work performed by M.R. Rosenzweig and others in the 1960s revealed major structural, cellular, and neurochemical differences between the brains of rats living in EE as compared to standard conditions (SC; Bennett et al., 1964). These, and multiple subsequent studies revealed a plethora of changes induced by exposure to a more challenging environment than the standard laboratory conditions which, arguably, may be characterized as a paradigm for sensory, motor, and social deprivation (Consorti et al., 2019; Rogers et al., 2019). Most of these changes converge on mechanisms increasing the adaptive plasticity of the brain, in line with the positive effects of EE on behavioral flexibility and experience-dependent learning (Donato et al., 2013; Ball et al., 2019; Gelfo, 2019). Of note, the cognitive-behavioral effects of EE include improved spatial memory formation (Leggio et al., 2005; Bennett et al., 2006; Eckert et al., 2010), highlighting the importance of hippocampal and neocortical networks which are, indeed, strongly affected by EE.

At the molecular and cellular level, effects of EE include increased expression of neurotrophic factors like BDNF (Novkovic et al., 2015; von Bohlen und Halbach and von Bohlen und Halbach, 2018), increased efficacy of synaptic transmission and plasticity (Ohline and Abraham, 2019; Cooper and Frenguelli, 2021), alterations in perineuronal nets (Sale et al., 2007), increased adult neurogenesis (Kempermann, 2019) and others. At the network level, changes in the number and function of inhibitory interneurons lead to changes in excitation-inhibition ratio, favoring increased activity of cortical principal cells (Sale et al., 2007; Greifzu et al., 2014). Finally, recordings from freely behaving rats have shown selective changes in multi-cellular representations of spatial contexts including increased sparsity and increased propensity to the remapping of place cells (Bilkey et al., 2017). Together, exposure of rodents to enriched environments results in multiple alterations at different system levels which, together, increase behavioral flexibility and adaptation. Importantly, genuine effects of an enriched environment can be differentiated from the effects of increased motor activity which, by itself, also induces multiple changes in cells and networks (Rogers et al., 2019).

Recent years have revealed much insight into the network-level functions supporting spatial memory formation and consolidation in rodents. A central concept is that memory formation is supported by highly specific spatio-temporal activity patterns of selected neurons. These patterns form on top of state-dependent network oscillations providing a temporal scaffold for the ordered spiking of neuronal ensembles (O’Keefe and Recce, 1993; Wilson and McNaughton, 1994; Buzsaki and Draguhn, 2004). Despite impressive progress in this field, there is, however, little information about alterations in hippocampal network oscillations by exposure to EE. We hypothesized that experience of an enriched physical and social environment is likely to induce alterations at this level which forms a critical link between cellular-molecular and cognitive-behavioral processes. We focused on a particular pattern of network activity in the hippocampus called sharp wave-ripple complexes (SPW-R). These complexes form propagating waves of increased synaptic activity superimposed by high-frequency oscillations at around 200 Hz (Buzsaki et al., 1992). Pyramidal cells fire with high precision within this fast rhythm, enabling re-activation of previously formed neuronal ensembles (Wilson and McNaughton, 1994; Ylinen et al., 1995; Lee and Wilson, 2002). These activity patterns are then transferred to the neocortex and are believed to support the consolidation of previously acquired spatial information (Buzsaki, 2015; Khodagholy et al., 2017).

In order to test for potential effects of EE on memory-related network patterns in the hippocampus, we exposed mice to an enriched environment for 10–15 days and subsequently studied spontaneous network activity in hippocampal slices *in vitro*. We report several characteristic alterations in sharp wave-ripple complexes compared to both, mice kept in standard conditions and in conditions with increased motor activity. Thus, an enriched environment alters coordinated activity patterns in memory-related hippocampal networks.

## Materials and Methods

### Animals

All experiments were performed with male C57/Bl6N mice (Charles River, Sulzfeld, Germany) in compliance with the Federation of European Laboratory Animal Science Association (FELASA) guidelines and were approved by the federal government of Baden-Württemberg (AZ G-30/17 and G-188/15). Mice were kept at 21–24°C and 40–60% relative humidity in Scantainers (Scanbur BK A/S, Denmark) with a 12-h light and dark cycle. Food and water were provided *ad libitum*.

### Enriched Environment Paradigm

**Figure 1:**
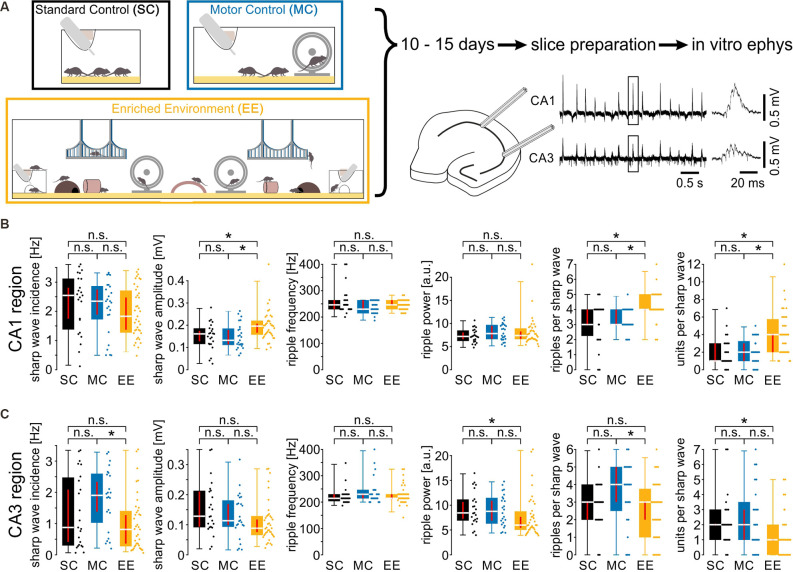
Environmental enrichment increases amplitude, ripple cycles, and unit activity during sharp wave-ripple oscillations. **(A)** Mice were kept in one of three housing conditions for 10–15 days: standard conditions, motor control, or enriched environment (for details, see “Materials and Methods” section). Subsequently, hippocampal slices were prepared to record local field potentials and unit discharges during spontaneous sharp wave-ripple oscillations. **(B)** Sharp wave amplitudes, ripples per sharp wave and units per sharp wave were increased in the CA1 region of slices from EE animals. **(C)** Activity in the CA3 region did not show any consistent alterations. *N* = 23 slices from six mice for standard conditions, *N* = 25 slices from five mice for motor control, and *N* = 39 slices from eight mice for environmental enrichment. Asterisks denote *p*-values < 0.05 from *post-hoc* testing followed by a significant Kruskal-Wallis test. Red lines depict the confidence intervals of the median assessed by bootstrapping. n.s., not significant.

Eight-week-old mice were housed for 10–15 days under three different conditions: Enriched Environment (EE), Motor Control (MC), or Standard Conditions (SC). Animals under SC and MC conditions were housed in groups of three in a standard-sized cage, whereas mice under EE conditions were kept as a group of 12 in a large cage sized 120 cm × 45 cm × 26 cm. Cages for standard conditions measured 27 cm × 22 cm × 14 cm and cages for motor control 36 cm × 24 cm × 19 cm. The EE cage (Figure 1A) was equipped with different kinds of stimuli, such as toys made of different types of materials (plastic tunnels, coconut caves, wooden bridges, and suspension bridges, running wheels, sticks), and several kinds of bedding (gravel, sand, mulch, and standard bedding). In order to control for increased physical activity in the EE group, mice in the MC group were provided with a running wheel. Mice that were taken out of the cage for experiments were not replaced.

### Preparation of Mouse Brain Slices

After the loss of the righting reflex under gradual CO_2_ anesthesia, the mice were rapidly decapitated and the brain was removed and stored in cooled (1–4°C), carbogen (95% O_2_/5% CO_2_) gassed, artificial cerebrospinal fluid (ACSF), containing in mM: 124 NaCl, 1.8 MgSO_4_, 1.6 CaCl_2_, 10 glucose, 1.25 NaH_2_PO_4_, 26 NaHCO_3_ (pH 7.4 at 37°C). The brain was then cut into 450 μm thick slices using a Leica VT1000S vibratome (Leica, Nussloch, Germany) and stored in a Haas-type interface recording chamber, where the slices were continuously perfused with oxygenated ACSF (1–2 ml/min) at 34 ± 1°C. Experiments were started after a recovery time of at least 3 h.

### Extracellular Recordings

Field potentials and unit discharges were recorded by lowering tetrodes made of four twisted tungsten wires (California Fine Wire, 12.5 μm diameter) into the pyramidal layer of the CA1 and the CA3 hippocampal region. The tetrodes were connected to an EXT-T2 amplifier (npi electronics). Signals were amplified 200×, low-pass filtered at 8 kHz, high-pass filtered at 0.3 Hz, and digitized at 20 kHz for off-line analysis (1401 ADC interface and Spike-2 data acquisition program, Cambridge Electronic Design CED, Cambridge, UK).

Analysis was done by custom routines written in Matlab (The MathWorks). Sharp waves were detected from low-pass filtered raw data (50 Hz) as local maxima with amplitudes larger than 0.12 mV within a 30 ms time window. This value corresponds to 4 SDs of event-free baseline noise (Both et al., 2008) yielding stable and reliable detection of SPW-Rs (as confirmed by visual inspection of traces and detected events). In order to assess potential differences in sharp wave incidence or properties, we included also events without visible superimposed ripples (see data points with 0 ripples per sharp wave in Figures 1B,C). Sorting sharp waves of each recording by amplitude and selecting the 50% largest events for analysis resulted in a data set of sharp waves which all contained ripples. Analysis of these data revealed qualitatively the same results, thus validating our unbiased approach.

Subsequently, SPW-R complexes were analyzed with continuous wavelet transform (complex Morlet wavelet, Both et al., 2008), starting 33 ms before and ending 67 ms after the peak of the detected sharp wave. From this wavelet spectrogram (50–300 Hz divided into 81 bins on a log scale), we extracted the leading ripple frequency and the peak power of the oscillation at frequencies >140 Hz. The number of ripple cycles of individual SPW-R were quantified from band-pass filtered raw data (140–250 Hz) and ripple troughs were detected as negative peaks exceeding three times the standard deviation of event-free baseline noise.

To detect extracellularly recorded action potentials, raw data were high-pass filtered at 500 Hz, and single events were extracted by setting a negative threshold at 4.5 SDs from background noise. Phase-coupling with respect to ripple oscillations was computed by assigning to each event a phase within one ripple cycle (see Figure 3). Ripple cycles were described as circular data of 360 degrees with ripple troughs set to zero degree. From these phases, the mean preferred firing angle and the precision were calculated. Precision is described by the length of the normalized mean vector (range 0–1). These parameters were determined for recordings with at least 33 SPW-R-associated action potentials which allowed for reliable calculation.

**Figure 2:**
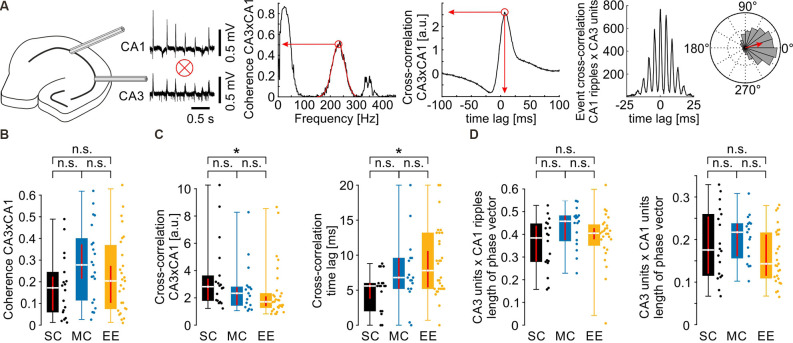
Propagation of network activity from CA3 to CA1 is not affected by environmental enrichment. **(A)** Schematics of the quantification of different parameters. For the normalized mean phase vector see also Figure 3A. **(B)** Coherence between CA3 and CA1 in the ripple frequency band was not different between groups. **(C)** Waveform cross-correlation between CA3 and CA1 showed differences between SC and EE but not between MC and EE or SC and MC. **(D)** CA3 unit to CA1 ripple (left) and CA3 unit to CA1 unit (right) phase coupling was not different after environmental enrichment. *N* = 23 slices from six mice for standard conditions, *N* = 25 slices from five mice for motor control, and *N* = 39 slices from eight mice for environmental enrichment. Asterisks denote *p*-values <0.05 from *post-hoc* testing followed by a significant Kruskal-Wallis test. Red lines depict the confidence intervals of the median assessed by bootstrapping. n.s., not significant.

**Figure 3:**
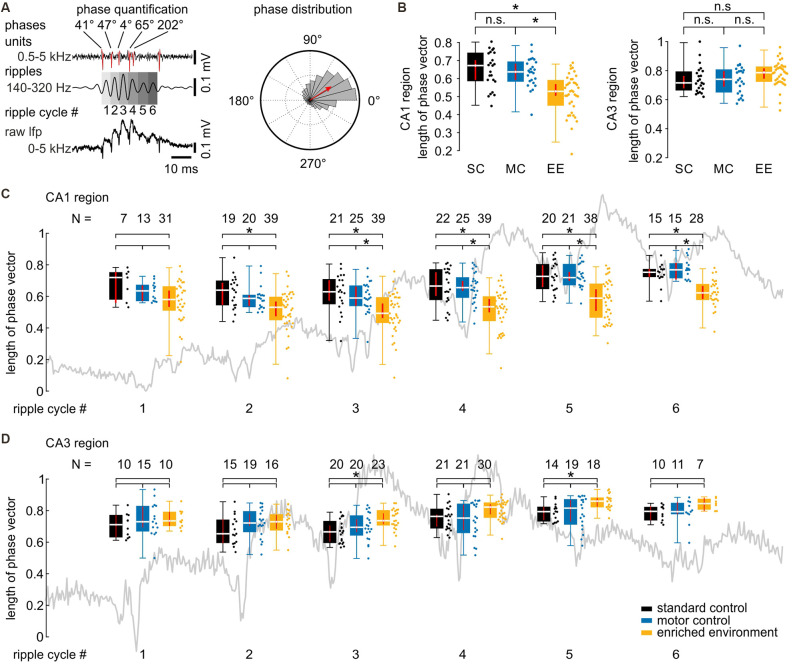
Coupling precision between spikes and field ripples is decreased after environmental enrichment. **(A)** Schematics of firing precision quantification. Each ripple cycle was divided into 360° starting with the first detectable ripple trough of a sharp wave-ripple complex. Spike time was measured as the phase within the respective ripple cycle (left panel, upper values). Precision was then quantified by the length of the normalized mean vector (red arrow in the right panel). **(B)** Firing precision is reduced in CA1 but not CA3 as can be seen by the decreased length of the phase vector. *N* = 23 slices from six mice for SC, *N* = 25 slices from five mice for MC, and *N* = 39 slices from eight mice for EE. **(C)** EE-induced differences in firing precision develop over the time course of SPW-R. **(D)** The CA3 region shows no differences in firing precision between EE and the two control groups. The N values above the graphs show the number of slices included in the analysis of the respective ripple cycle (minimum of 33 spikes, see “Materials and Methods” section; length of normalized mean vector in CA1 [median and {confidence interval}]: ripple cycle #1: SC: 0.72 {0.56, 0.76}; MC: 0.64 {0.57, 0.67}; EE: 0.58 {0.53, 0.64}; ripple cycle #2: SC: 0.64 {0.55, 0.69}; MC: 0.59 {0.54, 0.61}; EE: 0.53 {0.47, 0.57}; ripple cycle #3: SC: 0.63 {0.57, 0.70}; MC: 0.59 {0.54, 0.66}; EE: 0.49 {0.46, 0.55}; ripple cycle #4: SC: 0.67 {0.59, 0.75}; MC: 0.66 {0.61, 0.70}; EE: 0.53 {0.50, 0.58}; ripple cycle #5: SC: 0.73 {0.66, 0.80}; MC: 0.72 {0.70, 0.76}; EE: 0.59 {0.51, 0.65}; ripple cycle #6: SC: 0.75 {0.72, 0.77}; MC: 0.77 {0.71, 0.81}; EE: 0.62 {0.58, 0.67}). Asterisks denote *p*-values <0.05 from *post-hoc* testing following a significant Kruskal-Wallis test. Red lines depict the confidence intervals of the median assessed by bootstrapping. n.s., not significant.

### Whole-Cell Patch-Clamp Recordings

**Figure 4:**
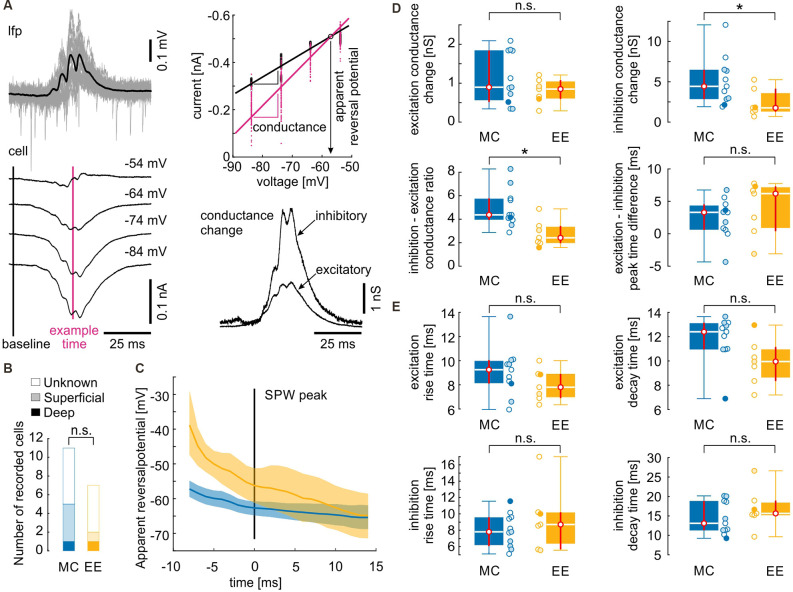
Inhibitory inputs during sharp waves are reduced after environmental enrichment. **(A)** Illustration of conductance analysis (similar to Borg-Graham et al., 1998; Haider et al., 2006). SPW-R were aligned by their peak (upper left panel, black line is the mean SPW-R). Currents from different holding potentials (lower left panel; mean currents from one recording) yielded instantaneous membrane conductance by fitting their I–E relation (upper right panel): black line is the linear regression fitted to the individual values 30 ms before the SPW peak. The slope of the linear regression yields the input conductance of the cell. Every subsequent time point can be similarly fitted. As an example, the time point near the SPW peak is depicted in magenta. The intersection with the baseline regression yields the apparent reversal potential of the conductance change. This allows a separation of the conductance change into an inhibitory and an excitatory component which is depicted in the lower right panel (see also “Materials and Methods” section). **(B)** From the 18 recorded cells, only seven locations could be recovered. However, there is no evidence that the MC group mainly consisted of superficial cells while the EE group mainly consisted of deep cells or *vice versa*. **(C)** Time course of the apparent reversal potential. Lines depict the median values, the shaded areas depict the 95% confidence intervals assessed by bootstrapping. Apparent reversal potential is shifted towards more positive values in EE cells at the ascending phase of the SPW. **(D)** Excitatory conductance changes and timing between excitatory and inhibitory inputs do not differ between control and environmental enrichment. However, inhibitory conductance changes and inhibitory–excitatory conductance change ratio is decreased after environmental enrichment. **(E)** Dynamics of conductance changes quantified as the time between the 20% and the 80% value did not change after environmental enrichment. Open circles depict cells with unknown locations, lightly filled circles depict cells from the superficial layer and filled circles depict cells from the deep layer. *N* = 11 cells from four mice for motor control and *N* = 7 cells from four mice for environmental enrichment. Asterisks denote *p*-values < 0.05 computed by a Mann–Whitney U test. Red lines depict the confidence intervals of the median assessed by bootstrapping. n.s., not significant.

For voltage clamp, glass pipettes (5–7 MΩ) were filled with an internal solution containing (in mM): 136 Cs-gluconate, 4 CsCl, 10 HEPES, 4 Mg-ATP, 0.3 Mg-GTP, and 10 Na_2_-phosphocreatine, adjusted to pH 7.3 with CsOH. For single cell staining and morphological analysis, cells were filled with biocytin (1–5%; Sigma-Aldrich #B4261). Blind patch-clamp recordings of pyramidal neurons in the hippocampal CA1 region were performed by lowering glass pipettes under continuous positive pressure into the cell layer until an increase in series resistance was observed. Subsequently, a seal was formed by applying light suction. After obtaining a gigaohm seal and breaking through the patch, the cell was clamped to −74 mV. Signals were recorded using a MultiClamp 700 B amplifier (Axon Instruments, Burlingame, CA) connected to a 1401 ADC interface (CED, Cambridge, UK) using Signal4 and Spike2 (v7) Software. To measure the conductance properties of the individual cells, we used the method described by Borg-Graham et al. (1998) and Haider et al. (2006). Under voltage clamp conditions, individual cells were held at five different membrane potentials (−84 mV, −64 mV, −54 mV, +16 mV) for 60 s separated by 10 s breaks at “resting” membrane potential (−74 mV) while simultaneously recording spontaneously occurring SPW-R oscillations. Ripples were then aligned by the peak of individual sharp waves, and input conductance G_in_(t) was calculated from the slope of the linear regression of the I-V relation between I(t) and V_holding_ (see Figure 4A). The apparent reversal potential E_rev_ was computed from the intersection between the linear regression at 0 s, i.e., at rest, and at time t. The change in conductance was taken as G_syn_(t) = G_in_(t) − G_in_(0). Inhibitory and excitatory conductances were then computed as G_syn,i_ = G_syn_ · (E_rev_ − *E*_AMPA_)/(E_GABA_ − E_AMPA_). G_syn,e_ = G_syn_ − G_syn,i_. E_AMPA_ was set to 0 mV and E_GABA_ to −75 mV.

### Immunohistochemistry and Confocal Microscopy

Under deep anesthesia (Ketamin/Xylazin, 120 and 16 mg/kg), mice were perfused with polychlorinated biphenyl (PCB) and subsequently with 4% paraformaldehyde (PFA). The perfused brain was removed from the skull and stored at 4°C in a 4% PFA solution. After fixation, the brain was cut into 50 μm thick slices using a vibratome (Leica VT 1200S). For antigen retrieval, slices were stored in a citrate-buffered phosphate-buffered saline (PBS) solution with a pH of 6.0 and kept in a compartment dryer at 100°C for 25 min. The slices were rinsed three times in PBS for 10 min before they were pretreated with a blocking solution containing 5% normal goat serum (NGS) in PBS for 1 h. After rinsing the slices (three times for 10 min), the primary antibodies (mouse anti-GAD67; 1:500; MAB5406, Merck Millipore; rabbit anti-PV; 1:1,000; PV 27, Swant) were applied and the slices were incubated overnight. The slices were again rinsed (three times for 10 min) and incubated in a PBS solution containing the secondary antibodies (Alexa Fluor 488 anti-rabbit; 1:1,000; A-11008, Molecular Probes by ThermoFisher Scientific; Cy3 anti-mouse; 1:500; 115-165-003, Dianova) for 2 h at room temperature. Subsequently, after rinsing the slices (three times for 10 min), a DAPI staining (1:10,000; Carl Roth, Germany) was performed for 2 min at room temperature. The slices were washed once again for 15 min and then mounted onto glass slides in Mowiol (81381, Sigma-Aldrich). The confocal images were acquired using a Nikon A1R point scanning confocal microscope (Nikon Imaging Center (NIC), Heidelberg University) with a 20× (0.75 NA) objective in air or a 60× (1.4 NA) oil immersion objective. The images were taken with a resolution of 1024 × 1024 pixels in z-stacks with a step size of 1 μm. After the acquisition, the images were analyzed in ImageJ/Fiji (Wayne Rasband, NIH, open source).

### Statistics

Data are represented as box plots in which medians are indicated by a line and the box limits denote the interquartile range (25%; 75%). Whiskers of box plots extend to the 2.5th and 97.5th percentile. Confidence intervals for the medians were estimated by means of bootstrapping, i.e., by computing the 2.5th and 97.5th percentile of the distribution of medians from 10,000 resamples. These confidence intervals are plotted as red vertical lines in the box plots. In the text, values are reported as medians and their confidence intervals. Statistical significance was calculated by the Kruskal-Wallis test for three groups, followed by a Dunn-Bonferroni *post hoc* test if testing reached significance or the Mann–Whitney U test for two groups. A p-value <0.05 was regarded as significant. To test for a potentially biased recording location of whole-cell patch clamp recordings we took the following approach: the location of seven out of 18 cells (5/11 MC cells and 2/7 EE cells) was histologically identified. Based on these data we computed the probability for a strong bias in the respective cell populations (i.e., 70% or more MC cells from one layer and 70% or more EE cells from the other layer). This was done by performing 100,000 simulations, assuming a 50% chance to record a cell from either layer. In a second approach, we substituted the 50% chance level by the observed percentages in histologically reconstituted cells (five superficial cells of seven in total). The probability of a strong bias was 1,028/100,000 cases (~1%) for 50% probability and 136/100,000 cases (~ 0.14%) for the 5/7 probability (see “Results” section).

## Results

### Environmental Enrichment Increases Amplitude, Ripple Cycles, and Unit Activity During Sharp Wave-Ripple Oscillations in CA1 but Not CA3

Mice were kept in one of three conditions: standard conditions with no additional stimuli (SC), standard cages with an added running wheel (“motor control”; MC), and enriched environment including enhanced social interactions (‘enriched environment’; EE) for 10–15 days (Figure 1A left panel). Subsequently, we recorded network-, multi unit- and cellular activity in acutely prepared hippocampal slices (see “Materials and Methods” section). Field potentials in CA1 and CA3, respectively, revealed spontaneous network events resembling sharp wave-ripple complexes (SPW-R, Maier et al., 2003, Figure 1A right panel). While there were no obvious changes in SPW-R waveforms, a quantitative analysis revealed several differences between the three conditions. In CA1, sharp wave amplitudes and the number of ripples per sharp wave were enhanced following an enriched environment (Figure 1B). Such differences were absent between animals with enhanced motor activity and standard conditions (SPW amplitudes [in mV, median and {confidence interval}]: SC: 0.16 {0.13, 0.17}; MC: 0.13 {0.11, 0.18}; EE: 0.20 {0.17, 0.21}; number of ripple cycles [per SPW]: SC: 3 {3, 4}; MC: 3 {3, 4}; EE: 4 {4, 4}; SC: *N* = 23 slices from six animals; MC: *N* = 25 slices from five animals; EE: *N* = 39 slices from eight animals). Sharp wave incidence, ripple frequency, and ripple power were not different between all three groups. Network patterns like sharp wave-ripple complexes provide a framework for the coordination of neuronal activity. We, therefore, extracted unit activity from the recordings and quantified the number of discharges per sharp wave. Following enriched environment conditions firing of units was clearly enhanced (Figure 1B, right panel; [per SPW]: SC: 1 {1, 3}; MC: 2 {1, 3}; EE: 4 {2, 4}). In contrast, recordings in CA3 did not reveal any consistent differences between enriched environment, motor control, and standard conditions (Figure 1C). Thus, the three different conditions revealed region-specific changes in coordinated network and unit activity during SPW-R.

### Propagation of Network and Unit Activity Is Not Altered by Environmental Enrichment

SPW-R are propagated from CA3 to CA1 (Both et al., 2008). We therefore tested whether environmental enrichment has an effect on this coordinated activity (Figure 2). Coherence between CA3 and CA1 in the ripple frequency band was not different between the three housing conditions (Figure 2B, SC: 0.17 {0.06, 0.22}; MC: 0.28 {0.21, 0.37}; EE: 0.20 {0.10, 0.27}). Similarly, there was no consistent change in cross-correlation between CA3 and CA1, despite small differences between standard control and environmental enrichment (Figure 2C, cross-correlation value: SC: 2.82 {1.83, 3.41}; MC: 2.34 {1.47, 2.79}; EE: 1.71 {1.39, 2.11}; cross-correlation time lag [ms]: SC: 5.6 {3.5, 6.0}; MC: 6.8 {5.2, 9.6}; EE: 7.8 {5.4, 10.6}). We next tested whether timing precision of units to the events propagating from CA3 to CA1was different between groups. Unit discharges in CA3 were correlated with the phase of the respective ripple cycle or unit discharges in CA1 as visualized in event cross-correlation histograms or circular plots (Figure 2A right, see also Figure 3A). To compensate for the delay during SPW-R propagation, time points of unit discharges and ripple troughs in CA1 were shifted by the time lag quantified by the cross-correlation of sharp waves between CA3 and CA1. Temporal firing precision was assessed by the normalized length of the summed phase vector. Neither CA3 unit to CA1 ripple coupling nor CA3 unit to CA1 unit coupling was altered by environmental enrichment (Figure 2D; CA3 unit × CA1 ripple phase vector length: SC: 0.38 {0.28, 0.44}; MC: 0.46 {0.37, 0.48}; EE: 0.40 {0.38, 0.43}; CA3 unit × CA1 unit phase vector length: SC: 0.18 {0.12, 0.25}; MC: 0.22 {0.16, 0.24}; EE: 0.14 {0.11, 0.21}).

### The Precision of Cell Firing Is Reduced Following Environmental Enrichment In CA1 but Not CA3

Temporal firing synchrony is not only propagated from CA3 to CA1 but is especially precisely synchronized within local networks in CA3 and CA1 (Ylinen et al., 1995; Both et al., 2008; Bahner et al., 2011). We, therefore, tested whether timing precision was different between groups, in addition to the increased unit firing rate in EE animals (Figure 3). Normalized length of the summed phase vector revealed a significant decrease in firing precision following enriched environment in CA1 but not in CA3 (Figure 3B; length of normalized mean vector in CA1 [median and {confidence interval}]: SC: 0.67 {0.59, 0.74}; MC: 0.64 {0.59, 0.68}; EE: 0.53 {0.50, 0.57}; CA3: SC: 0.72 {0.68, 0.76}; MC: 0.74 {0.69, 0.80}; EE: 0.79 {0.74, 0.82}; SC: *N* = 23 slices from six animals; MC: *N* = 25 slices from five animals; EE: *N* = 39 slices from eight animals). A more detailed analysis revealed that the loss in precision developed over time during each sharp wave: while there was no difference in spike precision on the initial ripple cycles, a pronounced reduction in the precision of neurons from the enriched environment group occurred during the second half of the sharp wave-ripple complex (Figure 3C). Again, CA3 did not show any alterations of unit precision on any ripple cycles (Figure 3D).

### Inhibitory Input but Not Excitatory Input During Sharp Wave-Ripples Is Reduced Following Environmental Enrichment

Previous work has revealed that propagating SPW-R involves strong synaptic excitation of CA1 pyramidal cells followed by pronounced, rhythmic perisomatic inhibition at ripple frequency (Ellender et al., 2010; Maier et al., 2011; Gan et al., 2017; Geschwill et al., 2020). We, therefore, analyzed the contribution of inhibitory and excitatory synaptic input during SPW-R in animals from enriched environments compared to motor controls. Standard conditions were not included in this analysis due to the absence of any differences to MC in the field- and unit activity. Whole-cell patch clamp recordings from CA1 pyramidal cells were performed at different holding potentials, aligned with SPW-R, and analyzed for the relative contribution of excitatory and inhibitory synaptic inputs, respectively; Borg-Graham et al., 1998; Haider et al., 2006; Figure 4A). Cells were recorded ‘blindly’ by inserting the patch clamp electrode into the stratum pyramidale guided by a low-magnification stereo microscope that was not able to resolve single cells. Therefore, recordings likely include neurons from both, the deep and the superficial pyramidal cell layer. PV basket cells are highly active during SPW-R and exert stronger inhibition on deep layer pyramidal cells compared to superficial neurons (Lee et al., 2014; Valero et al., 2015). Therefore, we aimed to assess the position of the recorded cells to exclude a possible bias between the two groups. From the 11 cells recorded after MC treatment, we recovered five cells (one deep, four superficial) and from the seven cells recorded after EE we recovered two cells (one deep, one superficial, Figure 4B). We then calculated the probability that both samples were highly different, with at least 70% superficial cells in the MC group (i.e., 8 out of 11) and at least 70% deep cells in the EE group (i.e., five out of seven). This strongly biased distribution has a probability of 1.0% (see “Materials and Methods” section). Taking into account the observed bias towards superficial cells (five superficial vs. two deep cells), the probability lowers to 0.14%. This control largely excludes that the observed differences between cells from MC and EE conditions are due to different locations of neurons between the samples.

We then calculated the apparent reversal potential (E_rev_) of mixed excitatory and inhibitory postsynaptic currents during SPW-R. E_rev_ of mice from the enriched environment group was significantly shifted towards more positive values on the ascending phase of the SPW-R as compared to the motor control group (Figure 4C). Consequently, peak conductance of inhibitory inputs was significantly reduced in slices from enriched environment while the apparent excitatory conductance change did not show any alterations (Figure 4D; inhibitory conductance change [nS, median and {confidence interval}]: MC: 4.4 {2.8, 6.5}; EE: 1.8 {1.3, 4.1}; excitatory conductance change: MC: 0.90 {0.52, 1.84}; EE: 0.85 {0.60, 1.08}; MC: *N* = 11 cells from four animals; EE: *N* = 7 cells from four animals). In consequence, the inhibition-excitation (I/E) ratio was reduced following environmental enrichment (MC: 4.4 {3.9, 5.8}; EE: 2.4 {2.0, 3.4}). Timing of excitatory and inhibitory conductance changes was not different between motor controls and enriched environment (Figure 4B; [in ms] MC: 3.3 {0.6, 4.6}; EE: 6.2 {0.4, 7.3}). To quantify the dynamics of inhibitory and excitatory conductance changes, we calculated the rise and decay times between 20% and 80% of the maximal conductance change. Similar to the peak timing, we did not observe any differences between the two groups (Figure 4E; excitation rise time [in ms] MC: 9.3 {8.1, 10.1}; EE: 7.8 {6.9, 8.9}; excitation decay time [in ms] MC: 12.4 {11.0, 13.2}; EE: 10.0 {8.4, 11.2}; inhibition rise time [in ms] MC: 7.8 {6.1, 9.6}; EE: 8.7 {5.7, 10.2}; inhibition decay time [in ms] MC: 13.1 {11.3, 18.9}; EE: 15.7 {15.2, 19.0}). Thus, inhibition during SPW-R is reduced after EE, in line with the increase in unit discharge rate.

### Neither the Number of Inhibitory Cells nor the Number of PV^+^ Inhibitory Cells Is Altered Following Environmental Enrichment

**Figure 5:**
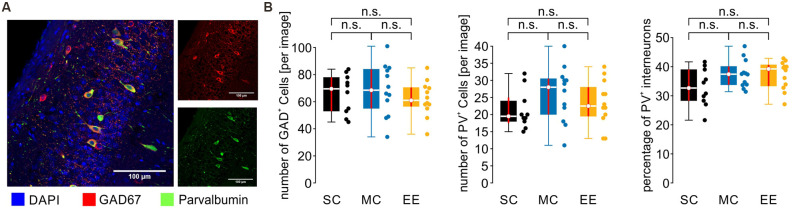
The number and distribution of inhibitory and PV^+^ inhibitory cells are not different between control animals and environmental enrichment. **(A)** Example image from CA1. Cell nuclei are stained by DAPI in blue, inhibitory cells by GAD67 in red, and PV^+^ inhibitory cells in green. **(B)** Neither the number nor the percentage of inhibitory and PV^+^ inhibitory cells is changed by environmental enrichment. *N* = 10 slices from five mice for standard conditions, *N* = 12 slices from six mice for motor control, and *N* = 12 slices from six mice for environmental enrichment. Red lines depict the confidence intervals of the median assessed by bootstrapping. n.s., not significant.

SPW-R go along with increased activity of inhibitory interneurons, particularly involving fast-spiking perisomatically inhibiting parvalbumin-expressing (PV^+^) neurons (Klausberger and Somogyi, 2008). In order to control for potential differences in the number of these inhibitory interneurons, we stained slices from all experimental groups for parvalbumin and the global GABAergic marker GAD67 (glutamate decarboxylase 67; Figure 5A). However, none of the quantifications revealed any significant differences between the cell populations or the ratio between PV^+^ and GAD67^+^ (Figure 5B; number of GAD67^+^ cells [per image, median and {confidence intervals}]: SC: 69.5 {51, 78}; MC: 68.5 {55, 84}; EE: 61 {56.5, 70.5}; number of PV^+^ cells: SC: 19.5 {17.5, 25}; MC: 28 {20, 30.5}; EE: 22.5 {19.5, 28}; percentage of PV^+^ cells: SC: 32.7 {28.3, 39.0}; MC: 37.4 {33.8, 40.1}; EE: 39.2 {33.3, 40.6}; SC: *N* = 10 slices from five animals; MC: *N* = 12 slices from six animals; EE: *N* = 12 slices from six animals). Thus, the different environmental conditions used in this study do not alter the number or percentage of PV^+^ interneurons in the hippocampus.

## Discussion

Environmental enrichment has been a mainstream paradigm in neuroplasticity research for several decades (Bennett et al., 1964; Consorti et al., 2019). Although there is much knowledge about the effects of EE at the molecular, cellular, and behavioral levels, much less is known about changes at the network level. We, therefore, analyzed memory-related network activity in the mouse hippocampus following exposure to EE for 10–15 days. We report that environmental enrichment in adult mice leads to an increase in network activity during sharp wave-ripple complexes (SPW-R) in CA1. At the cellular level, these changes are accompanied by decreased inhibition during sharp waves and with less precise firing during ripple oscillations. Thus, EE induces specific alterations of a spatio-temporal pattern of network activity which is involved in memory consolidation and retrieval (Lee and Wilson, 2002; Buzsaki, 2015; Khodagholy et al., 2017).

EE has profound effects on memory processing, including the formation, consolidation, and retrieval of spatial and declarative memory (Bilkey et al., 2017; Ohline and Abraham, 2019; Smail et al., 2020). The mechanisms underlying this cognitive function have been intensely studied during the past decades, including experience-dependent tuning of single neurons (e.g., place cells) and complex multi-neuronal patterns which are formed on top of coherent network oscillations (Wilson and McNaughton, 1994; Lee and Wilson, 2002; Frank et al., 2006; Klausberger and Somogyi, 2008). These patterns may be the network-level correlate of memories. We focused on SPW-R (Buzsaki et al., 1992) which are involved in the consolidation of memories after the initial formation of place-encoding patterns in the hippocampus (Buzsaki, 2015; Khodagholy et al., 2017). SPW-R contains local network oscillations of particularly high frequency (~200 Hz). Neurons in the rodent hippocampus fire action potentials with strong coupling to this fast rhythm, reaching firing precision in the ms range (Ylinen et al., 1995). Key features of SPW-R are present in spontaneous network activity in mouse hippocampal slices (Maier et al., 2003; Bahner et al., 2011), making them an ideal model system to test for subtle alterations in inhibition-excitation balance, event-related network activity, and firing precision.

We found several differences of SPW-R in CA1 between EE and the two control groups, MC and SC: (i) increased amplitude of sharp waves; (ii) increased firing of units during SPW-R; (iii) increased number of ripple cycles per sharp wave; (iv) decreased precision of firing with respect to individual ripple cycles; and (v) decreased inhibition-excitation ratio in CA1 pyramidal cells. While the mechanisms underlying SPW-R are not entirely clear, most of these changes can be coherently explained by reduced synaptic inhibition during the events. Converging evidence from different groups has shown that SPW-R is accompanied by strong activation of fast-spiking interneurons mediating potent perisomatic inhibition of pyramidal cells (Ylinen et al., 1995; Klausberger and Somogyi, 2008; Gan et al., 2017). The resulting inhibitory postsynaptic potentials generate a strong rhythmic signal at the population level which has been suggested to entrain pyramidal cells into the ripple cycles (Ylinen et al., 1995; Schlingloff et al., 2014; Gulyas and Freund, 2015). Experimental evidence and computer models suggest that electrical coupling between pyramidal cell axons (Draguhn et al., 1998; Schmitz et al., 2001; Traub et al., 2002, 2012) or supra-linear integration of excitatory inputs to pyramidal cell dendrites (Buzsaki et al., 1996; Memmesheimer, 2010; Jahnke et al., 2015) are important additional mechanisms contributing to spike-ripple coupling. While these models are not mutually exclusive, the crucial contribution of rhythmic inhibition to oscillating network patterns is undisputed.

As noted above, differences between EE and controls were most pronounced in CA1 while field potentials and unit discharges were not consistently changed in CA3. While the reasons underlying this discrepancy remain presently unknown, there are several relevant differences between both sub-networks: CA3 pyramidal neurons express extensive recurrent excitatory connections which are likely involved in the initiation of SPW-R (Miles and Wong, 1986; Buzsaki, 2015; Guzman et al., 2016); feedforward inhibition is strongly driven by inputs from mossy fibers in CA3 and by Schaffer collaterals and entorhinal cortex inputs in CA1 (Sun et al., 2017; Valero and de la Prida, 2018); in addition, the relative abundance of different interneuron subtypes differs between regions, including a lower incidence of VIP-positive interneurons in CA3 compared to CA1 (Botcher et al., 2014). The latter difference may be particularly important since VIP-positive interneurons inhibit parvalbumin-expressing interneurons which are strongly activated during SPW-R (Klausberger and Somogyi, 2008; Guet-McCreight et al., 2020). An enriched environment has been shown to decrease inhibition of hippocampal principal cells following changes in VIP interneuron-mediated innervation of PV-expressing interneurons (Donato et al., 2013). Due to the larger fraction of VIP-expressing interneurons, this mechanism might be more pronounced in CA1 compared to CA3, providing a potential explanation for the selective increase in amplitude and unit firing during SPW-R in CA1.

In line with the potential decrease in activation of fast-spiking interneurons in CA1 we observed a reduction in inhibitory conductance change during SPW-R in CA1 pyramidal cells following EE exposure. This might explain the increased rate of unit discharges during SPW-R. A potential confound is the diversity of hippocampal principal cells which have recently been shown to differ with respect to localization, expression of calbindin, and efficacy of synaptic inhibition (Valero et al., 2015). We controlled for a possible bias with respect to superficial vs. deep layer cells and to exclude this possibility for the present data. As a consequence of reduced synaptic inhibition, net excitatory input to pyramidal cells may be prolonged, in line with the larger number of ripple cycles. The increase in sharp wave amplitude is less easily explained. We have previously shown that this extracellular voltage transient in the pyramidal layer is mostly generated by inhibitory synaptic currents, at least in brain slices (Schonberger et al., 2014). Other suggestions, however, include a contribution of return currents from dendritic excitatory inputs which, in turn, would again be compatible with enhanced pyramidal cell activity following reduced inhibition (Ylinen et al., 1995).

The precise mechanisms underlying the reduced inhibition during SPW-R remain presently elusive. We did not find any evidence for decreases in the total number of GABAergic cells or in the relative abundance of PV^+^ interneurons. Thus, it is likely that functional changes at inhibitory synapses or reduced recruitment of fast-spiking interneurons underlie the present observation. Indeed, previous work shows that EE alters the recruitment of interneurons and, hence, activity-dependent inhibition (Sale et al., 2007; Baroncelli et al., 2010; Eckert et al., 2010; Fu et al., 2015). The underlying mechanisms may include a reduction in perineuronal nets around inhibitory synapses (Kwok et al., 2011; Cattaud et al., 2018). In any case, the net decrease in inhibition during SPW-R is in line with most changes observed at the level of field potentials and unit discharges. This mechanism may be useful for developing new therapeutic strategies to increase synaptic plasticity in clinical situations like amblyopia (Consorti et al., 2019) or intellectual disability (Fernandez and Garner, 2007; Begenisic et al., 2011).

Motor activity has profound effects on neuronal network activity and plasticity (Voss et al., 2013; Ohline and Abraham, 2019). An enriched environment is expected to trigger increased motor activity which, hence, may account partially for EE-induced changes (van Praag et al., 1999; Donato et al., 2013; Vivar et al., 2013). We, therefore, included a second control group in our study, where mice were offered a running wheel in their home cages. These mice showed no consistent alterations in all examined parameters at the field potential level compared to controls in standard housing conditions. It is, thus, likely that our present findings are specific for environmental enrichment, possibly including a larger spectrum of sensory inputs, social interactions, or other factors. We do not exclude that the motor activity itself induced changes at the cellular level which we did not examine for standard controls. Indeed, such changes have been shown in the motor cortex (Donato et al., 2013) but also in the hippocampus where increased motor activity promotes adult neurogenesis and learning (van Praag et al., 1999, 2005; Bolz et al., 2015). We focused, however, on memory-related network activity which was not altered by a mere increase in motor activity. At the network level, our present analysis was restricted to SPW-R which are mostly involved in memory consolidation. Previous work, however, has shown that gamma oscillations, which are involved in memory encoding, are also enhanced by EE (Shinohara et al., 2013). Together, the resulting changes of network activity may contribute to the specific cognitive effects of EE, especially those related to spatial or declarative memory processing (Donato et al., 2013; Ball et al., 2019; Gelfo, 2019). Revealing such EE-induced changes at the network level may instruct future attempts to increase neuronal plasticity or mnemonic functions in neuro-psychiatric disorders where EE has already been shown to exert beneficial effects (Nithianantharajah and Hannan, 2006; Rogers et al., 2019).

In summary, our results show specific changes of spatio-temporal activity patterns and neuronal excitability during SPW-R following a short (10–15 days) exposure to an enriched environment. These changes are in line with, and potentially mediated by, a decrease in synaptic inhibition of pyramidal cells during SPW-R.

## Data Availability Statement

The raw data supporting the conclusions of this article will be made available by the authors, without undue reservation.

## Ethics Statement

The animal study was reviewed and approved by Regierungspräsidium Karlsruhe, 76247 Karlsruhe.

## Author Contributions

MB and AD conceived and designed the experiments. LL, MK, and DH performed experiments. LL, MK, DH, and MB analyzed the data. MB, AD, and LL wrote the original manuscript. All authors revised and edited the manuscript. All authors contributed to the article and approved the submitted version.

## Conflict of Interest

The authors declare that the research was conducted in the absence of any commercial or financial relationships that could be construed as a potential conflict of interest.

## Publisher’s Note

All claims expressed in this article are solely those of the authors and do not necessarily represent those of their affiliated organizations, or those of the publisher, the editors and the reviewers. Any product that may be evaluated in this article, or claim that may be made by its manufacturer, is not guaranteed or endorsed by the publisher.
